# Supplementary material for: Pemafibrate Induces a Low Level of PPARα Agonist-Stimulated mRNA Expression of ANGPTL4 in ARPE19 Cell
Source: Bioengineering (Basel). 2024 Dec 9;11(12):1247. doi: 10.3390/bioengineering11121247 (PMC11673482; doi:10.3390/bioengineering11121247)

Fig. S1. M-A plot (A) and volcano plot (B) for ARPE19 cells not treated with a PPARα agonist (NT) vs ARPE19 cells treated with pemafibrate (Pema).

Two-dimensionally cultured ARPE19 cells not treated with a PPARα agonist (NT cells, n=3) and those treated with 10 μM of pemafibrate (Pema cells, n=3) were loaded to RNA sequencing analysis. Differentially expressed genes (DEGs) are shown by an M–A plot (A) and a volcano plot (B). Colored points represent either overexpressed (red) or underexpressed (blue) DEGs in NT cells compared with those in Pema-treated cells.

Figure S1A M-A plot NT vs Pema

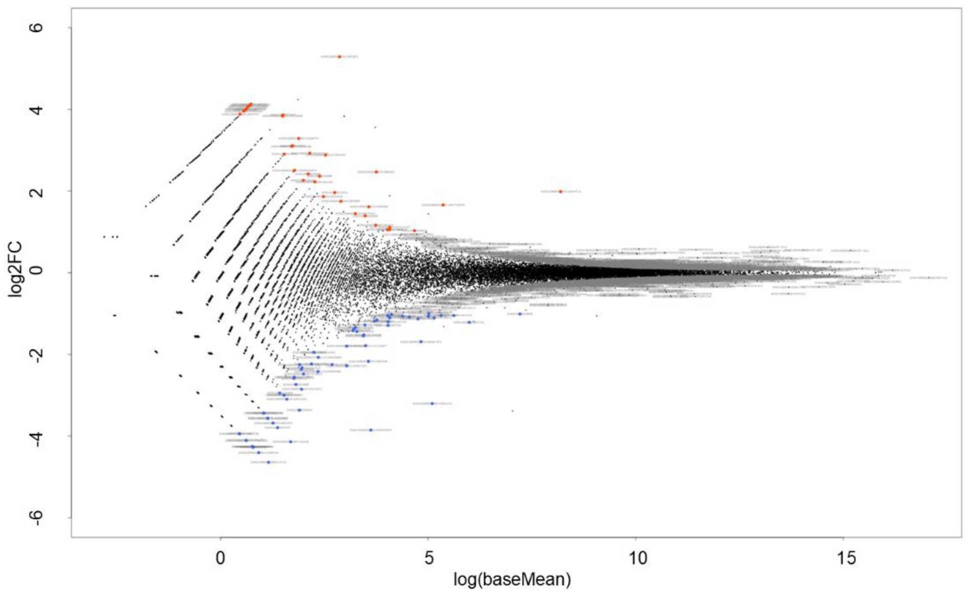

Figure S1B volcano plot NT vs Pema

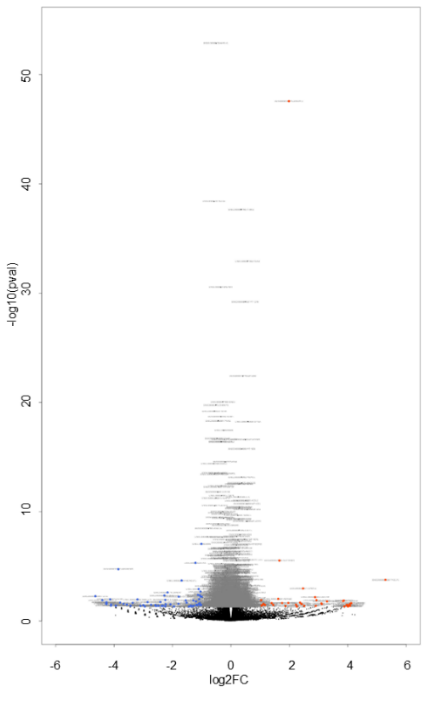

Fig. S2. M-A plot (A) and volcano plot (B) for ARPE19 cells not treated with a PPARα agonist (NT) vs ARPE19 cells treated with GW7647 (GW).

Two-dimensionally cultured ARPE19 cells not treated with a PPARα agonist (NT cells, n=3) and those treated with 20 μM of GW7647 (GW cells, n=3) were loaded to RNA sequencing analysis. Differentially expressed genes (DEGs) are shown by an M–A plot (A) and a volcano plot (B). Colored points represent either overexpressed (red) or underexpressed (blue) DEGs in NT cells compared with those in GW-treated cells.

Figure S2A M-A plot NT vs GW

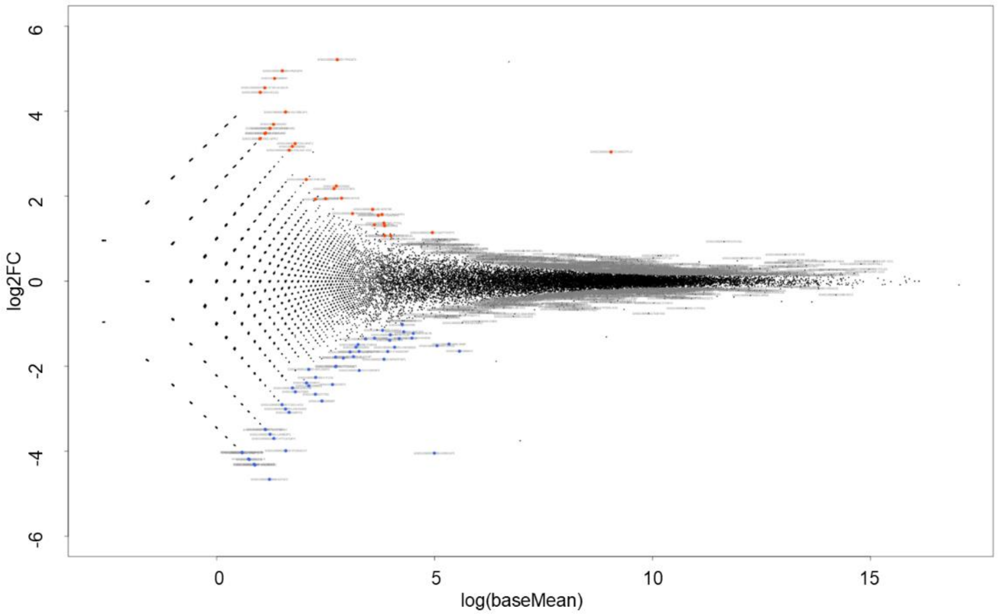

Figure S2B volcano plot NT vs GW

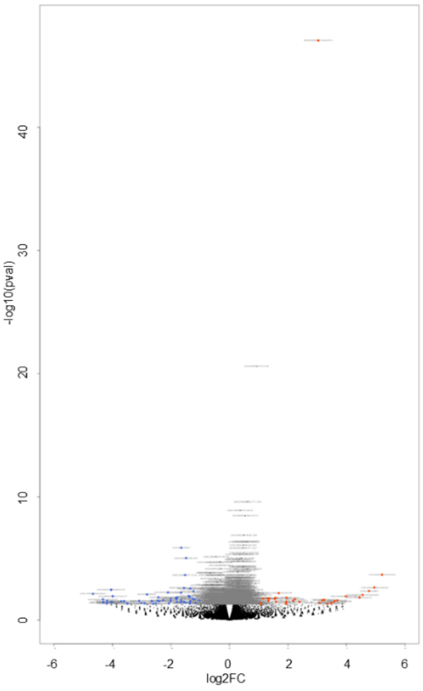

Fig. S3. M-A plot (A) and volcano plot (B) for ARPE19 cells treated with pemafibrate (Pema) vs ARPE19 cells treated with GW7647 (GW).

Two-dimensionally cultured ARPE19 cells treated with 10 μM of pemafibrate (Pema cells, n=3) or 20 μM of GW7647 (GW cells, n=3) were loaded to RNA sequencing analysis. Differentially expressed genes (DEGs) are shown by an M–A plot (A) and a volcano plot (B). Colored points represent either overexpressed (red) or underexpressed (blue) DEGs in Pema-treated cells compared with those in GW-treated cells.

Figure S3A M-A plot Pema vs GW

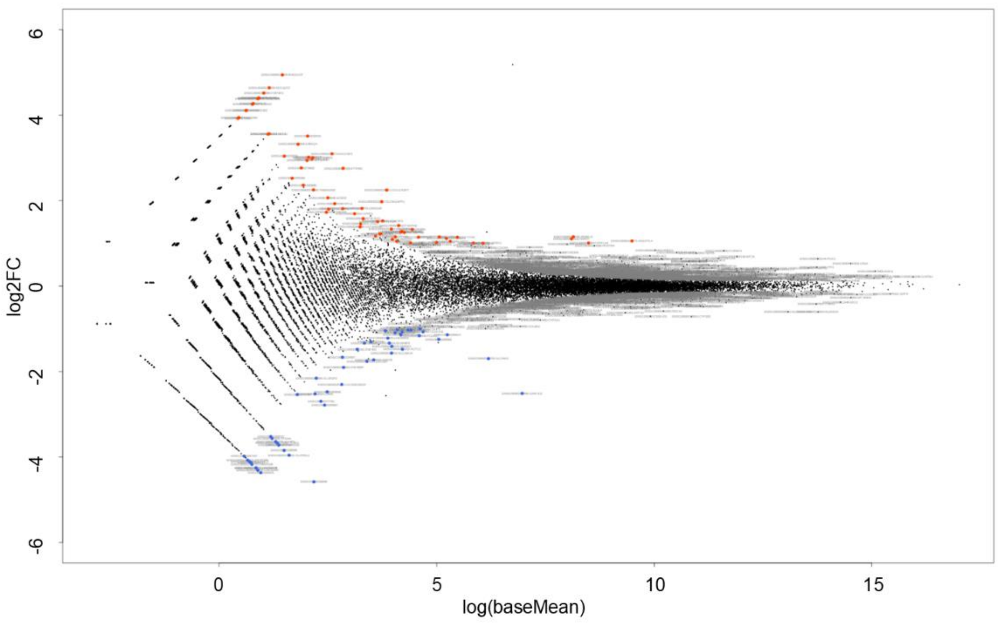

Figure S3B volcano plot Pema vs GW

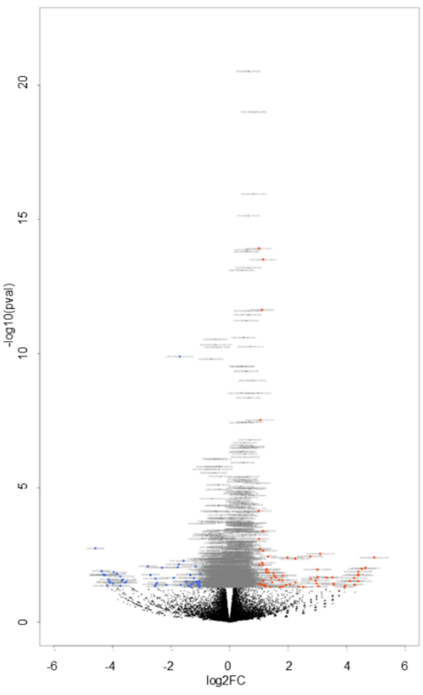

Fig. S4. DEGs categorized by cell component-related factors by GO enrichment analysis.

Two-dimensionally cultured ARPE19 cells not treated with a PPARα agonist (NT cells, n=3) and those treated with 10 μM of pemafibrate (Pema cells, n=3) or 20 μM of GW7647 (GW cells, n=3) were loaded to RNA sequencing analysis. GO enrichment analysis of DEGs was performed between NT vs Pema (panel A) and NT vs GW (panel B) and categorized by cell component-related factors. Bar color represents *p* values and the x-axis represents numbers of DEGs.

Figure S4A cell component: NT vs Pema

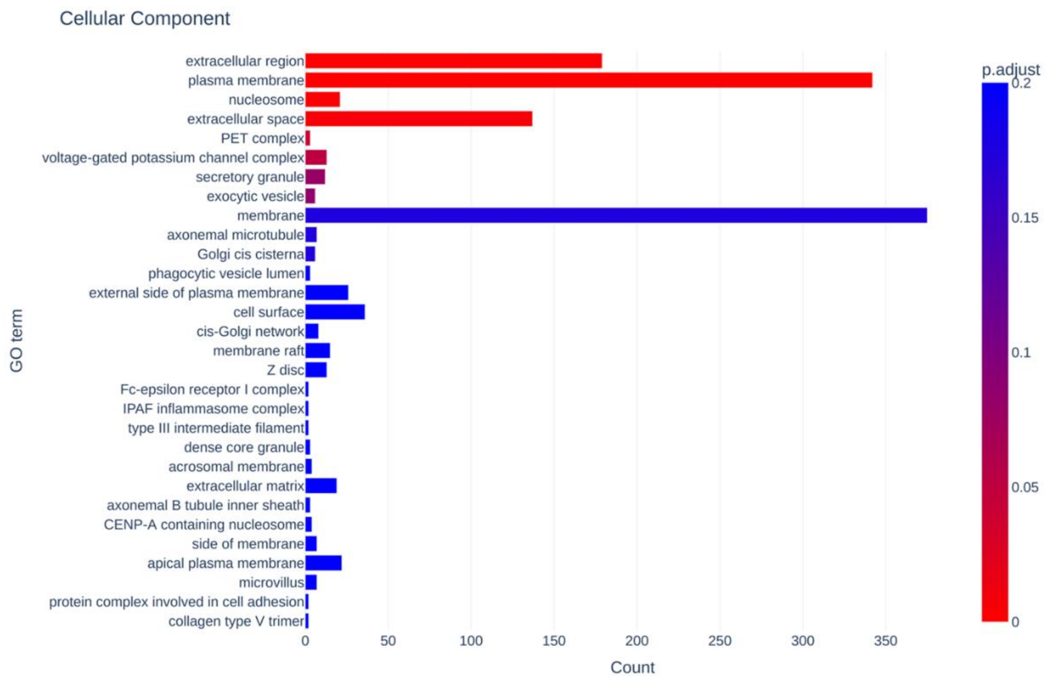

Figure S4B cell component: NT vs GW

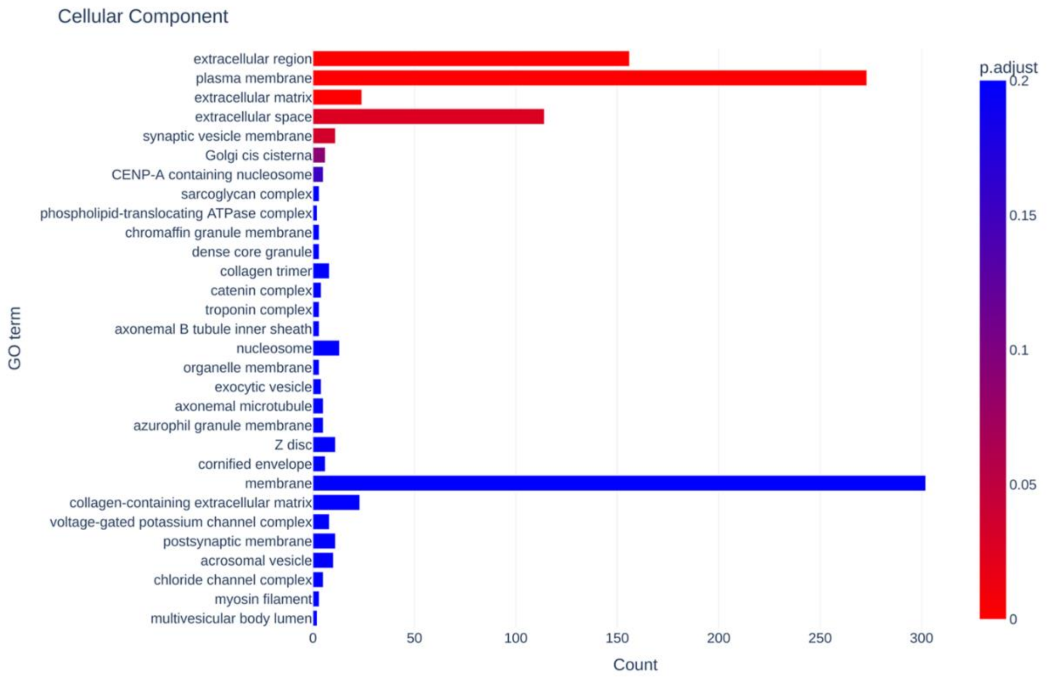

Fig. S5. DEGs categorized by molecular function-related factors by GO enrichment analysis.

Two-dimensionally cultured ARPE19 cells not treated with a PPARα agonist (NT cells, n=3) and those treated with 10 μM of pemafibrate (Pema cells, n=3) or 20 μM of GW7647 (GW cells, n=3) were loaded to RNA sequencing analysis. GO enrichment analysis of DEGs was performed between NT vs Pema (panel A) and NT vs GW (panel B) and categorized by molecular function-related factors. Bar color represents *p* values and the x-axis represents numbers of DEGs.

Figure S5A Molecular function: NT vs Pema

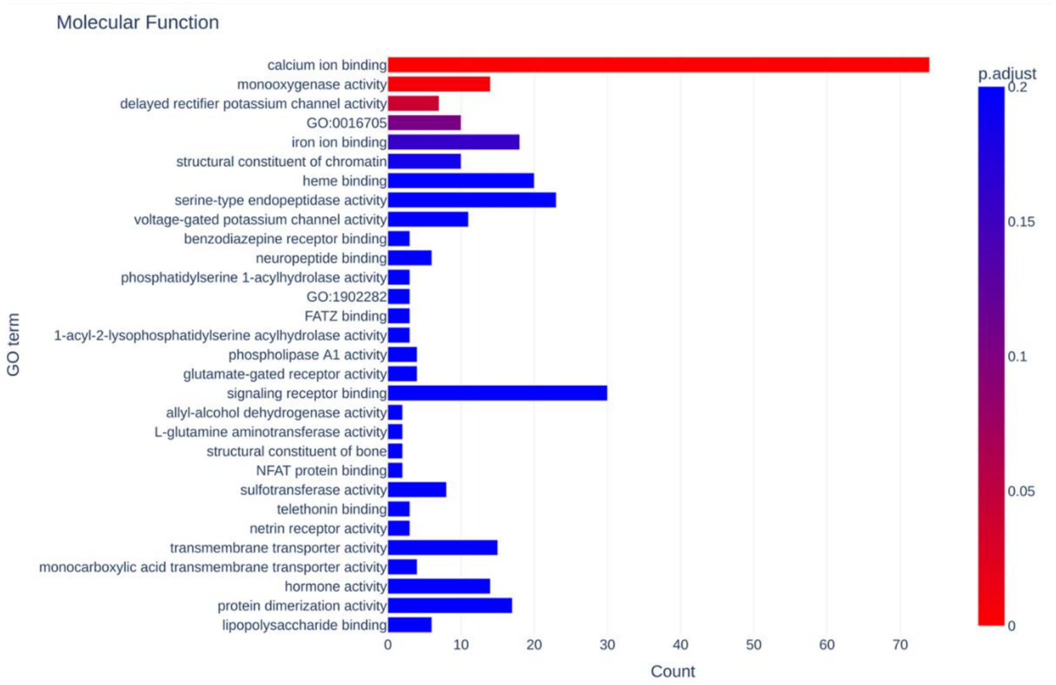

Figure S5B Molecular function: NT vs GW

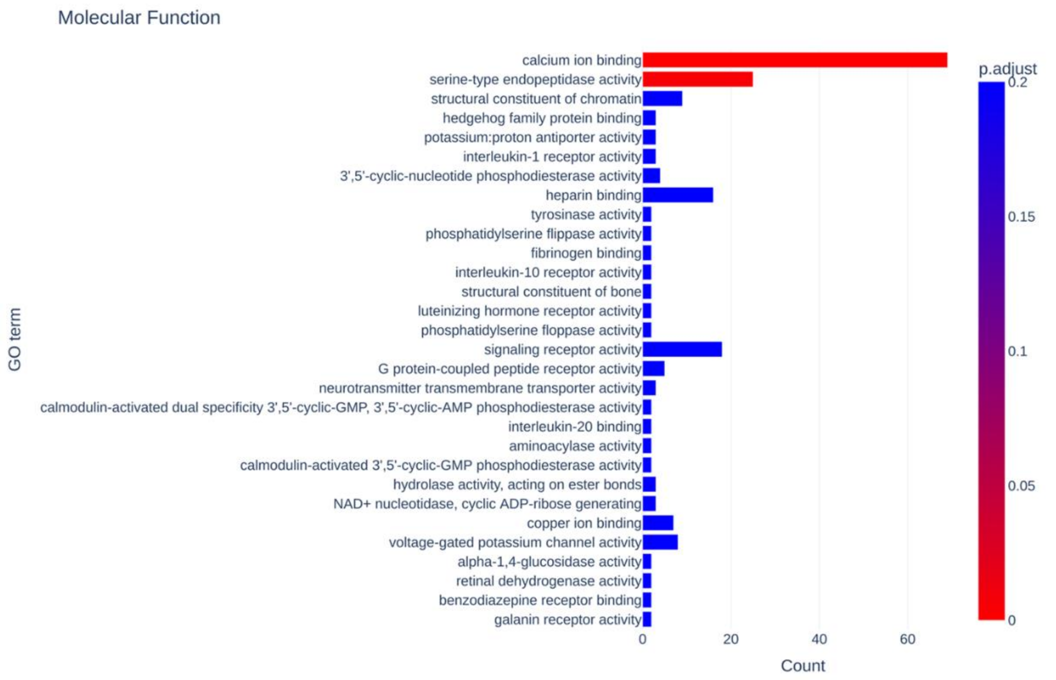

Fig. S6. DEGs categorized by biological process-related factors by GO enrichment analysis.

Two-dimensionally cultured ARPE19 cells not treated with a PPARα agonist (NT cells, n=3) and those treated with 10 μM of pemafibrate (Pema cells, n=3) or 20 μM of GW7647 (GW cells, n=3) were loaded to RNA sequencing analysis. GO enrichment analysis of DEGs was performed between NT vs Pema (panel A) and NT vs GW (panel B) and categorized by biological process-related factors. Bar color represents *p* values and the x-axis represents numbers of DEGs.

Figure S6A Biological process: NT vs Pema

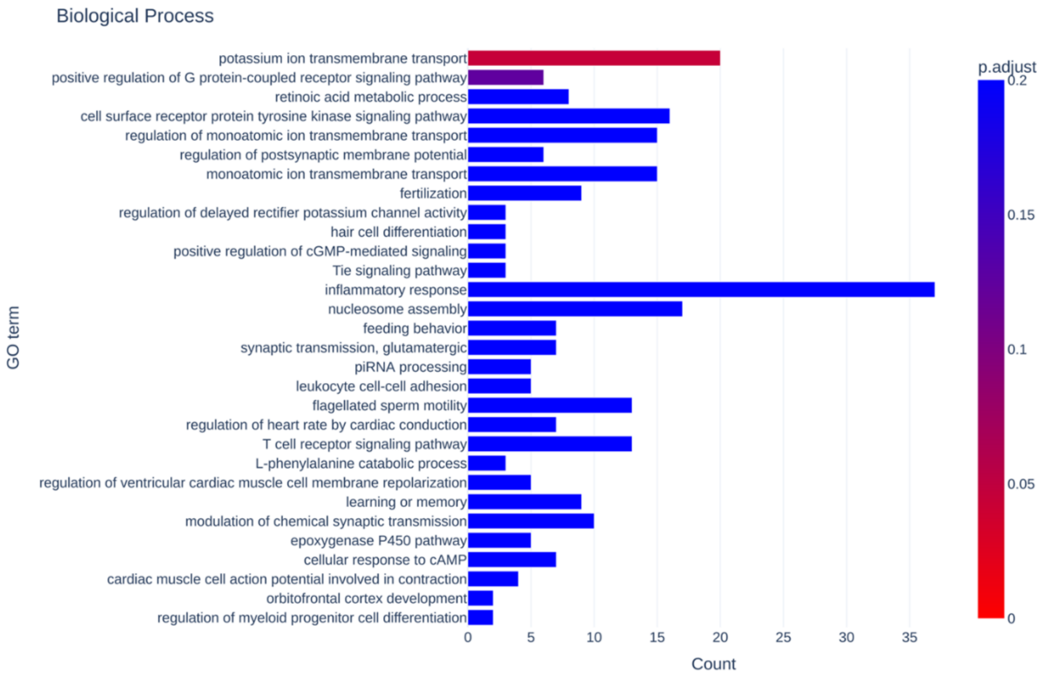

Figure S6B Molecular function: NT vs GW

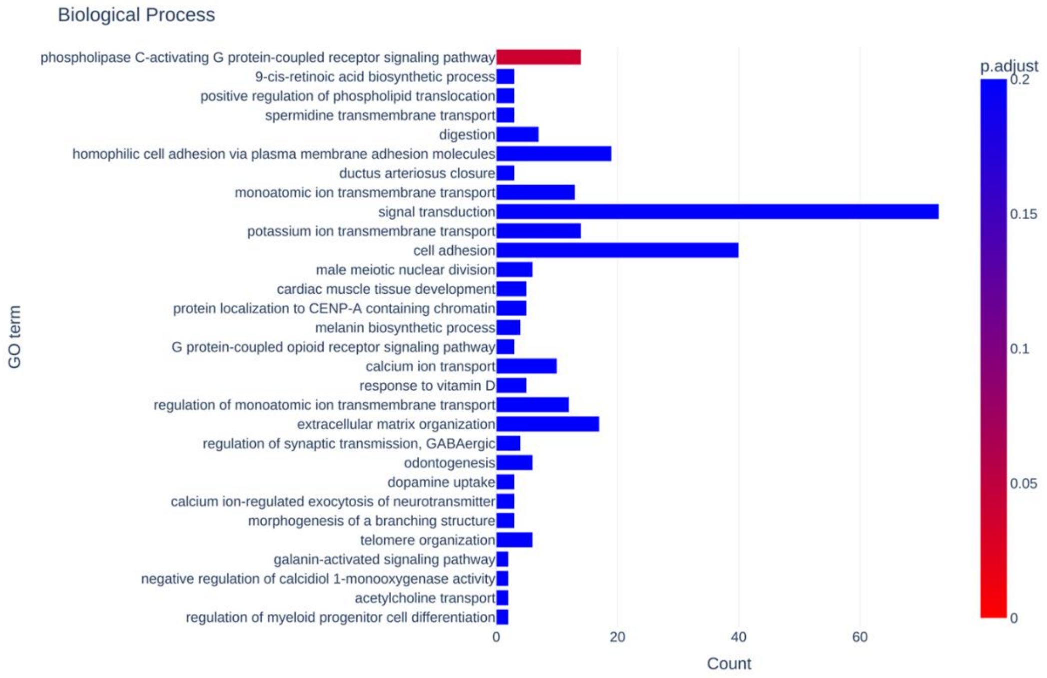

Supplement: Supplementary file 1 [file bioengineering-11-01247-s001.zip › Pema Supplemental.pdf]
